# Use of Nerve Transfer Procedures for Motor and Sensory Restoration of a High Median Nerve Injury

**DOI:** 10.7759/cureus.26205

**Published:** 2022-06-22

**Authors:** Abelardo Medina

**Affiliations:** 1 Surgery, University of Mississippi Medical Center, Jackson, USA

**Keywords:** high median nerve injury, motor deficit, sensory deficit, nerve repair, nerve transfer, nerve grafting

## Abstract

High median nerve (HMN) injuries are unusual clinical conditions, but they generate significant disability of the affected extremities to perform even basic activities of daily living. Even though they can display different degrees of dysfunction due to overlapping innervation and musculature compensation, an early assessment of the existing functional deficits and a timely surgical approach can optimize the long-term outcome. The use of distal nerve transfer procedures has gained popularity since they reduce the distance between the injured zone and the disrupted targets, accelerate the nerve regeneration and subsequently optimize the postoperative motor and sensory recovery.

This report describes a patient with a significant segmental loss of the median nerve at the upper third of the left arm after a motor vehicle accident that caused multiple other injuries. The motor deficit of this injury was managed soon after the admission with extensor carpi radialis brevis (ECRB) nerve transfer to the anterior interosseous nerve (AIN). Subsequently, double side-to-side cross-palm nerve allografts between the ulnar and median nerves were utilized to restore the sensory deficit of the HMN lesion.

An important functional improvement was obtained with these nerve transfer procedures, and the patient successfully returned to the workforce without limitations. Other surgical options for motor and sensory reconstruction are briefly reviewed.

## Introduction

High median nerve (HMN) injuries are defined by their location above the origin of the anterior interosseous nerve (AIN). These lesions can produce a significant impact on the affected extremities and deteriorate the patients’ capacity to work and even perform basic activities of daily living. Even though different degrees of clinical findings may occur due to overlapping innervation and musculature compensation, the traditional description of these injuries includes lack of forearm pronation, weakness of wrist flexion, loss of index and long fingers’ flexion, absence of sensation of radial half of the palm and ring finger as well as volar aspect of the thumb, index and middle fingers [1].

The timing for nerve repair and the distance between the injured level and the targeted structures (e.g., muscles, skin) are crucial for a meaningful functional outcome. In this regard, distal nerve transfer procedures shorten the reinnervation process, and therefore optimize the postoperative motor and sensory recovery [2]. Thus, nerves from expendable muscles can be transferred to denervated distal stumps of transected nerves. For instance, extensor carpi radialis brevis (ECRB), brachialis, abductor digiti minimi (ADM), and supinator are among the most frequently utilized donor sites of motor branches [1,3,4]. In addition, the distal transfer of sensory nerves can provide protective sensation to highly functional areas such as pulps of thumb and index finger. Thus, the dorsal sensory branches of the radial nerve going to the ulnar side of the thumb and radial side of the index finger can be transferred to their corresponding proper digital nerves [5]. The cross-palm nerve graft procedure with either autograft or acellular allograft nerves has also been described for sensory restoration [6].

## Case presentation

**Figure 1:**
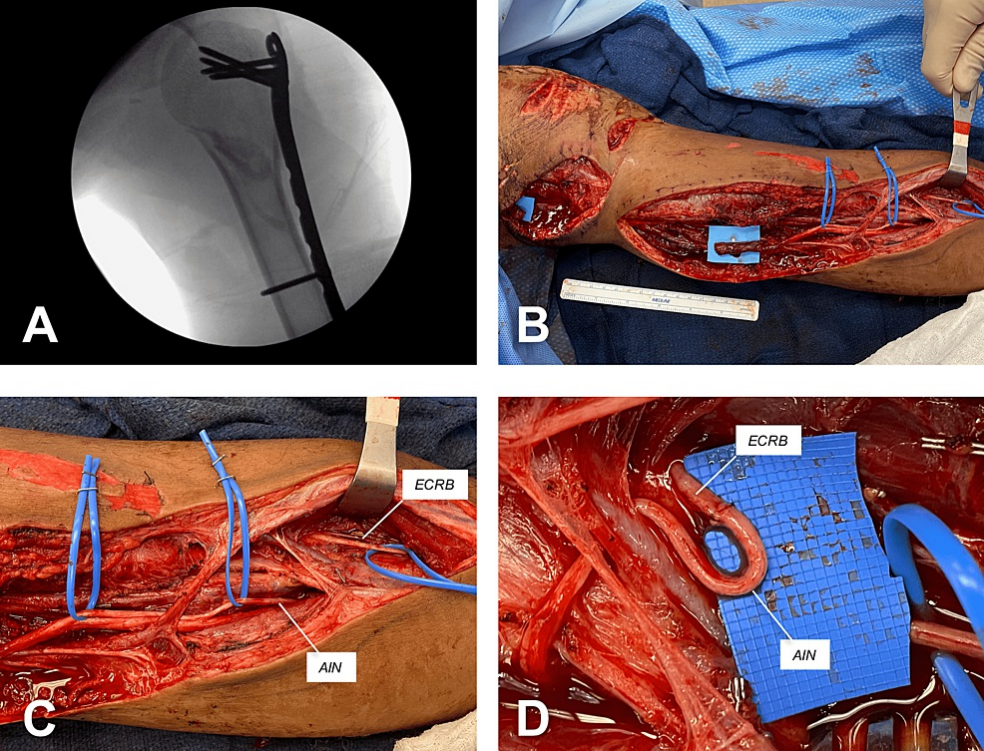
Initial surgical reconstruction of left upper extremity injuries. (A) X-ray showing bony stabilization with a proximal humerus locking plate. Interposition vein graft for brachial artery transection was also performed. (B) Median nerve transection with stumps nearly 15 cm apart. (C) Dissection of extensor carpi radialis brevis nerve (ECRB) and anterior interosseous nerve (AIN). (D) ECRB-to-AIN transfer by performing end-to-end coaptation with four simple interrupted stitches of 8-0 nylon. The coaptation site was subsequently wrapped with a nerve protector.

An 18-year-old male unrestrained driver was involved in a motor vehicle rollover. The accident caused multiple injuries including moderate left pneumothorax, comminuted fracture in the left distal ulnar diaphysis, fracture of greater trochanter of the left femur, and comminuted open fracture of the proximal left humerus associated with a complete transection of the brachial artery. The injuries of the left upper extremity required an urgent bony stabilization with a proximal humerus locking plate and arterial repair with a reversed great saphenous vein graft (Figure 1, panel A). An extensive gap of the median nerve (MN) at the upper third of the left arm was also noticed during this initial surgery.

Our initial local examination revealed no flexion of index finger and thumb along with weak middle finger flexion and thumb opposition. No sensation over the classic MN territory was also found. Forearm pronation and wrist flexion were not examined due to the presence of splint for ulnar fracture immobilization. Five days after the admission, the surgical repair of the MN injury was performed without a tourniquet. Following debridement of the wound bed and careful dissection around the previously placed interposition vein graft, the proximal and distal stumps of the MN transection were found nearly 15 cm apart (Figure 1, panle B). Subsequently, the original incision on the upper medial arm was extended caudally in a step-like fashion. Using blunt dissection and bipolar electrocautery, the fasciotomy of the arm and forearm along the MN trajectory was completed. After lateral mobilization of brachioradialis muscle, the superficial branch of the radial nerve (SBRN) and the extensor carpi radialis brevis (ECRB) nerve were identified. Distally, the ECRB nerve had two branches entering the ECRB muscle and their functional integrity was confirmed with a nerve stimulator. The dissection progressed in caudal direction to identify the AIN at its exit point on the radial aspect of the median nerve. Thereafter, extensive neurolysis of the AIN and MN was carried out in cephalad direction to maximize the AIN length for nerve transfer and examine the integrity of the distal MN stump (Figure 1, panels B and C). At this point, it was confirmed that the MN had an additional 10 cm of non-viable segment at its distal end. Due to the magnitude of the MN gap (±25 cm) and after the unhealthy nerve segment was sharply removed with the No. 10 blade, an ECRB-to-AIN transfer was performed as previously described [4]. Thus, the AIN was extensively freed and tunneled radially and posteriorly towards the ECRB nerve. The nerve coaptation was performed with four simple interrupted stitches of 8-0 nylon (Figure 1, panel D). A nerve protector was wrapped around the coaptation site and secured with 8-0 nylon sutures. The cleaned stumps of the MN transection were buried deep in the surrounding tissues and anchored with 6-0 prolene. Finally, after profuse irrigation of the wound bed with normal saline, the wound was closed in two layers, deep interrupted stitches of 3-0 vicryl followed by horizontal mattress stitches of 3-0 prolene to the skin.

**Figure 2:**
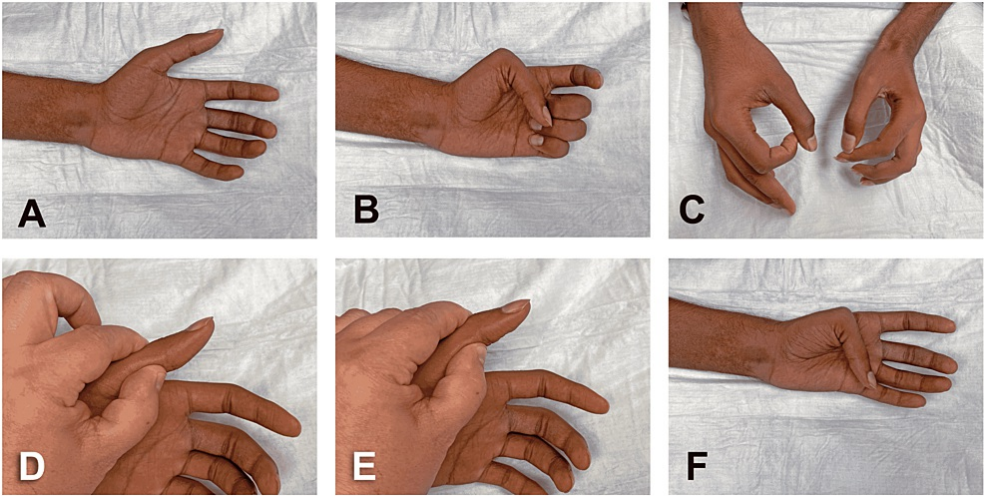
Three months after initial nerve transfer procedure. (A) Hand in supine position lying on the examination table and patient with eyes closed. (B) Hand fist showing no flexion of IP joint of the thumb (with partial thumb opposition), weak flexion of PIP joint of index finger, and no flexion of its DIP joint. (C) Weak and incomplete “okay sign.” (D) IP joint of the thumb in neutral position. (E) Weak and incomplete flexion of IP joint of the thumb (F) Kapandji’s thumb opposition score of 9 out of 10. IP: interphalangeal; PIP: proximal interphalangeal; DIP: distal interphalangeal

Three months after the surgery, the patient exhibited preliminary improvement of the motor deficits namely increase of range and motion and strength of thumb and index finger (Figure 2, panels A-F). The hand fist was weak with partial flexion of the proximal interphalangeal (PIP) joint of the index and minimal flexion at the IP joint of the thumb and distal IP joint of the index (Figure 2, panels B, D, and E). The flexion of the middle and ring fingers was present (Figure 2, panel B). The “okay sign” was feeble and incomplete (Figure 2, panel C). Using Kapandji’s test, the thumb opposition was almost comparable to the opposite side, but this range of motion was likely unrelated to the nerve transfer procedure (Figure 2, panel F) [1].

**Figure 3:**
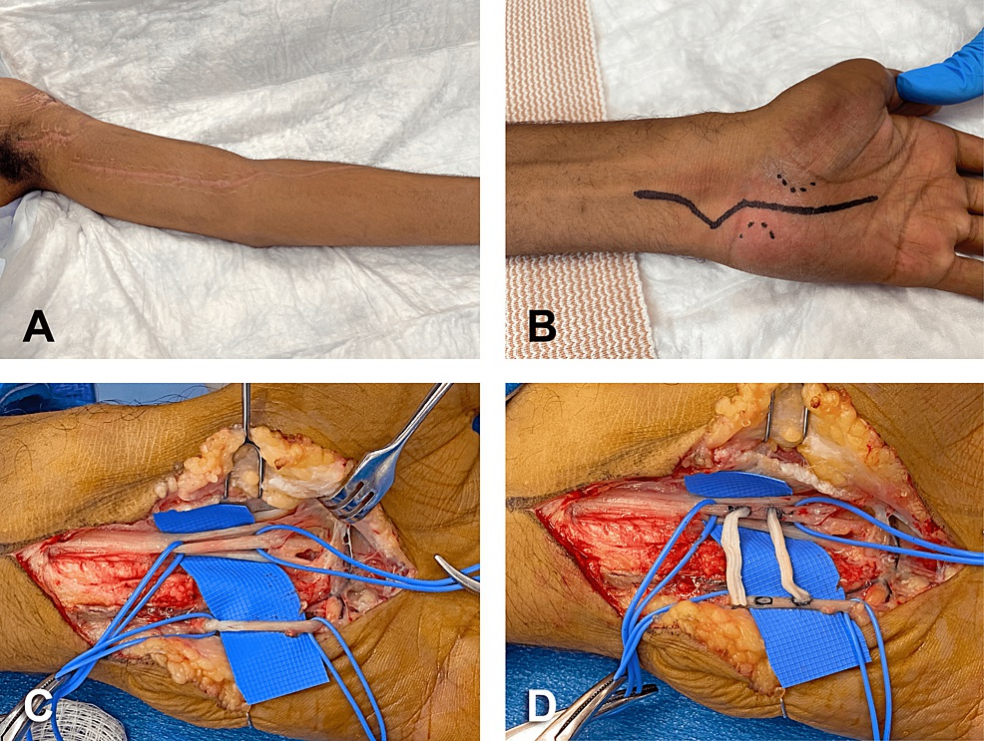
Secondary surgical reconstruction, four months post-injury. (A) Satisfactory healing process of original traumatic wounds and surgical incision. (B) Surgical approach of cross-palm nerve allograft procedure between the ulnar and median nerves. (C) MN intermediate sensory component going to the first and second webspaces (recipient) and the main sensory component of the ulnar nerve (donor) were at a distance of 1.8 cm for the double cross-palm nerve allografts. (D) Epi/perineural windows were created and side-to-side coaptation of both allograft cables was performed with six interrupted stitches of 9-0 nylon. MN: median nerve

Forearm pronation and wrist flexion were moderately restricted. The sensory deficit remained mostly unchanged since the accident. For this reason, the patient was brought back to the operating room. A cross-palm nerve allograft procedure between the ulnar and median nerves was performed to restore, at least in part, the sensory function of the MN [6]. At that point in time, the traumatic wounds and surgical incision from the previous intervention were in an advanced healing process (Figure 3, panel A).

Under tourniquet insufflation at 250 mmHg, the incision of the skin was made ulnar to the thenar crease and extended in a zig-zag fashion across the wrist (Figure 3, panel B). Thereafter, opening of the Guyon’s canal was carried out followed by identification of ulnar artery and nerve. Careful microdissection of the ulnar nerve (UN) was then accomplished to identify its motor branch, main sensory branch, and branch going to the hypothenar eminence. The motor branch was followed deep in the palm and released from surrounding hypothenar muscles and fascia by detaching them from the hook of the hamate. Next, neurolysis of the main sensory branch was conducted in proximal direction to get better separation from the motor component. The transverse carpal ligament and the nearby antebrachial fascia were divided to widely release the MN in the carpal tunnel. The recurrent motor branch of the MN was identified going to the thenar muscles. Then, extensive interfascicular neurolysis of the third webspace and recurrent components was carried out to separate them from the intermediate sensory component going to the first and second webspaces (Figure 3, panel C). Thus, the MN intermediate sensory component (recipient) and the main sensory component of the UN (donor) were at a distance of 1.8-cm for the double cross-palm nerve allografts. Two 2-cm cables of nerve allografts (3 mm in diameter) were selected for the procedure. Two epi/perineural windows were created on the surface of the sensory branch of MN (ventral aspect proximal window and ulnar aspect the distal window). Thereafter, two windows were created on the surface of the sensory branch of the UN (proximal and distal on ventral aspect). Side-to-side coaptation of both allograft cables was performed with 6 interrupted stitches of 9-0 nylon (Figure 3, panel D). Thereafter, fibrin glue was applied to seal the coaptations. At this point, the tourniquet was deflated and the hemostasis was achieved with bipolar electrocautery. For postoperative pain control, median nerve and ulnar nerve blocks were performed with a mix of 1% lidocaine and bupivacaine (half and a half) without epinephrine. Finally, the closure of the wound was done with multiple interrupted vertical mattress stitches of 3-0 nylon. 

**Figure 4:**
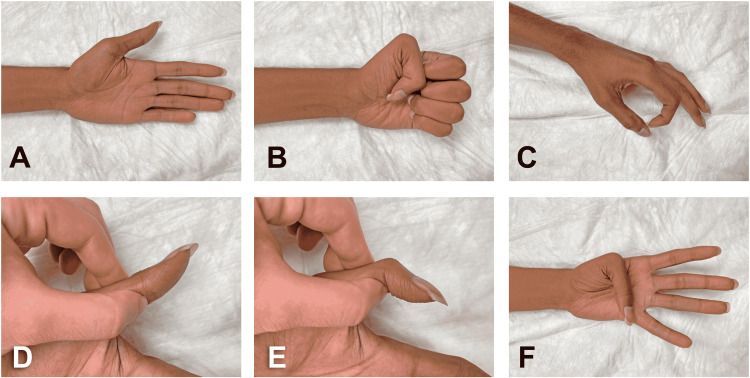
Long-term follow-up, 24 months post-injury. Range of motion demonstrating adequate MN motor recovery. (A) Hand in supine position lying on the examination table and patient with eyes closed. (B) Hand fist showing negative benediction sign. (C) “Okay” sign. (D) IP joint of the thumb in neutral position. (E) Flexion of the IP joint of the thumb. (F) Kapandji’s thumb opposition score of 10 out of 10. IP: interphalangeal

The patient was recently examined in our clinic 24 months after the ECRB-to-AIN transfer and 20 months after the cross-palm nerve allograft procedure. The patient stated that, since his previous appointment (12 months earlier), he has been working as a truck driver without problems and restrictions. He exhibited no evidence of hypertrophic scars or soft tissue edema (Figure 4, panel A).

On examination, the patient presented no local pain and significant improvement of range of motion and strength (Figure 4, panels B-F). Thus, normal flexion at the interphalangeal (IP) joint of the thumb and at the proximal and distal IP joints of the index finger were found. The thumb presented normal opposition (according to Kapandji’s score) and better abduction and pronation (Figure 4, panel F). A mildly weak forearm pronation and wrist flexion were noticed and attributed, at least in part, to the comminuted fracture of the distal ulna that was managed conservatively. Grasp and lateral pinch appeared weaker compared to these of the contralateral hand. All these motor deficits were causing no disruptions to the patient’s activities of daily living. The patient also experienced important postoperative sensory recovery after cross-palm nerve allograft procedure between the ulnar and median nerves. Using Semmes-Weinstein monofilaments as previously reported by Bertelli et al., an equivalent sensation to contralateral hand was detected on the entire thumb and palm including first webspace and thenar eminence [5]. However, the patient presented protective sensation (2-g monofilament) on the index finger up to the distal IP joint. No sensation was detected on the remaining MN territory with either 2-g or 4-g monofilaments. A 15-mm two-points discrimination appeared positive on thumb and MN territory of the palm including first webspace and thenar eminence. Unfortunately, the patient felt one point on the remaining distal MN territory of the palm and index finger. No sensation was perceived by the patient on the middle finger and MN territory of the ring finger. The topical application of ethyl chloride induced a cold sensation on the thumb, palm, and index finger up to the proximal IP joint. This sensation was equivalent to that on the contralateral hand. The patient reported no cold perception on the remaining MN territory. Of note, the double cross-palm nerve allografts’ procedure caused no sensory deficit on the UN territory.

## Discussion

The HMN injuries are unusual lesions of the upper extremities that exhibit, contrary to the classic description, significant variability of clinical deficits [7]. In general, these patients present no flexion of the IP joint of the thumb and the proximal and distal IP joints of the index finger. They have a severely decreased grasp and pinch strength and lack of sensation of the tips of the thumb and middle finger as well as proximal and middle phalanx of the index finger. Interestingly, the middle finger flexion, thumb opposition, and sensation of the palm are usually preserved [1,5,7]. Variable loss of forearm pronation, preservation of wrist flexion, and lack of sensation along the first webspace have also been reported [8]. HMN injuries exhibit poor functional recovery after the repair of injured sites regardless of the surgical approach. Accordingly, in our case, the nerve grafting was not considered a reasonable therapeutic option due to the length of the nerve graft required to bridge the existing gap and the significant distance between the level of injury and the site of the distal motor and sensory targets. Instead, distal nerve transfer procedures were selected to reduce the distance to the targets, and therefore, avoid inflammatory or fibrotic environments at the zone of injury, accelerate the reinnervation process and restore the function of motor or sensory targets in a timely manner [2]. Thus, within the recommended post-injury period of 10 months, an ECRB-to-AIN nerve transfer was utilized to restore index finger and thumb flexion, and in part increase the grasp and pinch strength [1]. Accordingly, during a long-term follow-up, the patient showed considerable recovery of range of motion and strength on the thumb and index finger. An additional transfer of the abductor digiti minimi (ADM) nerve to the thenar branch of MN could have been done in our patient to enhance his grasp and pinch weakness [1]. The opposition of the thumb and the flexion of the middle finger were not significantly affected by the high MN transection. Furthermore, even though he presented a deficit in forearm pronation and wrist flexion, they could have been associated with the underlying ulnar fracture. Regardless of the contributing factors, these restrictions have progressively improved during the post-injury period (24 months), and currently, they are causing no limitations to the patient’s activities. For sensory restoration, a double side-to-side sensory nerve grafting from UN to MN in the palm was performed. This procedure induces sensory axonal sprouting through the nerve allografts into the denervated MN sensory branch without causing damage to the donor UN sensory component [6]. During the long-term follow-up, the patient presented partial sensory recovery, especially on the thumb, first webspace, and proximal index. He reported no restrictions on his activities of daily living and at work secondary to this sensory deficit. An alternative procedure for the sensory reconstruction of this patient could have been the transfer of the dorsal digital nerves of the thumb (ulnar side) and index finger (radial side) to the corresponding palmar digital nerves. This very distal sensory nerve transfer has the additional advantage of reducing the risk of faulty location and neuropathic pain seen in more proximal procedures [1].

In summary, the reported patient underwent two distal nerve transfer procedures to restore motor and sensory functions after a high median nerve injury with extensive structural gap. After 24 and 20 months since these surgical interventions, the patient has recovered enough motor and sensory functions that allow him to have a highly demanding occupation without restrictions. Interestingly, however, it is difficult to estimate the definite contribution of nerve transfer techniques in the motor and sensory recovery of HMN injuries due to the presence of overlapping innervation that usually occurs below the elbow level [1]. Thus, the degree of recovery after nerve transfers can be partially explained by compensatory function re-education. For instance, motor (e.g., Riche-Cannieu and Marinacci anastomoses) and sensory (e.g., Berrettini anastomosis) connections between UN and MN distal to the elbow [8-10] may account for the preservation and/or functional improvement seen in pronation, middle finger flexion, wrist flexion, thumb opposition, and non-anatomic sensation loss, among others [1]. Therefore, the early assessment of existing motor and sensory deficits is crucial to establish the appropriate surgical approach in a timely manner.

## Conclusions

Distal nerve transfers are effective surgical procedures for HMN injuries that help optimize motor and sensory recovery that otherwise are not possible with the repair at the injured level. To get their maximum benefits, they should be done within 10 months post-injury. The surgical plans should be specific to the functional deficits and the patients’ expectations, and therefore they may consist of one or more procedures.
